# 51 The Association Between Neighborhood Disadvantage and Patient-Reported Outcomes in Burn Survivors

**DOI:** 10.1093/jbcr/irae036.075

**Published:** 2024-04-17

**Authors:** Arushi Biswas, Zachary H Zamore, Zohra V Aslami, Rafael Felix P Tiongco, Ayman Ali, Carisa M M Cooney, Mark Fisher, Julie A Caffrey, Sheera F Lerman

**Affiliations:** Johns Hopkins University School of Medicine, Baltimore, MD; Tulane University School of Medicine, Baltimore, Maryland; Duke Hospital, Durham, North Carolina; Johns Hopkins University, Baltimore, Maryland; Johns Hopkins School of Medicine, Baltimore, Maryland; Johns Hopkins University School of Medicine, Baltimore, MD; Tulane University School of Medicine, Baltimore, Maryland; Duke Hospital, Durham, North Carolina; Johns Hopkins University, Baltimore, Maryland; Johns Hopkins School of Medicine, Baltimore, Maryland; Johns Hopkins University School of Medicine, Baltimore, MD; Tulane University School of Medicine, Baltimore, Maryland; Duke Hospital, Durham, North Carolina; Johns Hopkins University, Baltimore, Maryland; Johns Hopkins School of Medicine, Baltimore, Maryland; Johns Hopkins University School of Medicine, Baltimore, MD; Tulane University School of Medicine, Baltimore, Maryland; Duke Hospital, Durham, North Carolina; Johns Hopkins University, Baltimore, Maryland; Johns Hopkins School of Medicine, Baltimore, Maryland; Johns Hopkins University School of Medicine, Baltimore, MD; Tulane University School of Medicine, Baltimore, Maryland; Duke Hospital, Durham, North Carolina; Johns Hopkins University, Baltimore, Maryland; Johns Hopkins School of Medicine, Baltimore, Maryland; Johns Hopkins University School of Medicine, Baltimore, MD; Tulane University School of Medicine, Baltimore, Maryland; Duke Hospital, Durham, North Carolina; Johns Hopkins University, Baltimore, Maryland; Johns Hopkins School of Medicine, Baltimore, Maryland; Johns Hopkins University School of Medicine, Baltimore, MD; Tulane University School of Medicine, Baltimore, Maryland; Duke Hospital, Durham, North Carolina; Johns Hopkins University, Baltimore, Maryland; Johns Hopkins School of Medicine, Baltimore, Maryland; Johns Hopkins University School of Medicine, Baltimore, MD; Tulane University School of Medicine, Baltimore, Maryland; Duke Hospital, Durham, North Carolina; Johns Hopkins University, Baltimore, Maryland; Johns Hopkins School of Medicine, Baltimore, Maryland; Johns Hopkins University School of Medicine, Baltimore, MD; Tulane University School of Medicine, Baltimore, Maryland; Duke Hospital, Durham, North Carolina; Johns Hopkins University, Baltimore, Maryland; Johns Hopkins School of Medicine, Baltimore, Maryland

## Abstract

**Introduction:**

Burns are a leading cause of morbidity and mortality, with long-term complications including pain and poor physical function. While neighborhood disadvantage has been shown to be associated with burn severity, its relationship with long-term burn complications has not yet been investigated. Therefore, we hypothesized that higher area of deprivation index (ADI), an aggregate marker of neighborhood disadvantage, will be associated with increased likelihood of pain and poor physical function post-burn.

**Methods:**

We obtained data from the Burn Model System (BMS) database. We linked patient data with ADI state decile 1-10 (1=least and 10=most disadvantaged) using year of injury and Census Block Group based on residence at time of injury. We used bivariate analyses to identify associations between ADI and patient and burn characteristics. We used multivariate regression to determine whether ADI was associated with self-reported pain and physical function (PROMIS-29 subscales) 6 and 24 months post-burn. Significance was p < 0.05.

**Results:**

We included 780 patients who were admitted to one of three BMS centers between 2014 and 2021 of whom 538 (69%) were male. Most identified as White (83%), Black (9.8%), or Asian (2.5%). Mean age at time of burn was 46.2 years, mean ADI state decile was 5.75, and mean TBSA was 15.2%. Bivariate analysis demonstrated significant associations between ADI and age at injury, ethnicity, race, pre-injury household income, pre-injury education, increased pain intensity at 6- and 24-month follow-ups, and worse physical function at 24-month follow-up. Multivariate regressions controlling for TBSA, race, age, sex, anxiety, depression, and pain interference demonstrated that ADI was a significant predictor of higher pain intensity at 6- (p=0.008) and 24- (p=0.020) month follow up and worse physical function at 24-month follow up (p=0.011).

**Conclusions:**

Burn patients living in areas of higher neighborhood disadvantage are more likely to report increased long-term pain and reduced physical function, even after controlling for other known risk factors. Further investigation into the relationship between neighborhood disadvantage and post-burn outcomes in larger, nationally representative samples is warranted.

**Applicability of Research to Practice:**

Along with existing literature, our findings suggest that policy targeting neighborhood disadvantage may help improve short- and long-term post-burn outcomes and assist in identifying patients who are at increased risk for negative health-related outcomes.

